# Recombinant BCG-*Ag85A* enhances antitumor immunity and controls melanoma progression through multimodal immune activation in mice

**DOI:** 10.1007/s00203-026-05093-0

**Published:** 2026-08-05

**Authors:** Suzana Lemke Lanius, Bruna Silveira Pacheco, Fernanda Severo Sabedra Sousa, Amilton Clair Pinto Seixas Neto, Nicole Ramos Scholl, Stella Julli Farias Cardozo, Maria Eduarda Ehlert, Valentina Gessinger Ferreira, Tiago Veiras Collares, Sibele Borsuk, Odir Dellagostin, Thais Larre Oliveira Bohn, Fabiana Kömmling Seixas

**Affiliations:** 1 Molecular and Cellular Oncology Research Group, Cancer Biotechnology Laboratory, Postgraduate Program in Biotechnology, Technological Development Center, Federal University of Pelotas, Pelotas, RS 96010900 Brazil; 2 Laboratory of Infectious and Parasitic Biotechnology, Postgraduate Program in Biotechnology, Technological Development Center, Federal University of Pelotas, Pelotas, RS Brazil; 3 Vaccinology Laboratory, Postgraduate Program in Biotechnology, Technological Development Center, Federal University of Pelotas, Pelotas, RS Brazil

**Keywords:** Melanoma, Bacillus Calmette-Guérin (BCG), Recombinant BCG, *Ag85A*, *Mycobacterium bovis*, Cancer immunotherapy, Prophylactic vaccination, Cancer vaccine

## Abstract

Melanoma is an aggressive form of skin cancer characterized by high metastatic potential and increasing incidence and mortality worldwide. Although significant advances have been achieved with immune checkpoint inhibitors and targeted therapies, effective preventive and long-term therapeutic strategies remain limited. Bacillus Calmette-Guérin (BCG), widely used in bladder cancer immunotherapy, exhibits potent immunostimulatory properties and has emerged as a promising platform for recombinant cancer vaccines. Among the candidate antigens, Antigen 85A (Ag85A), derived from *Mycobacterium bovis*, is notable for its strong immunogenicity and ability to induce robust Th1-mediated cellular immune responses. In this study, recombinant BCG strains overexpressing efficacy was evaluated in melanoma models *in vitro* and *in vivo*. Gene expression analysis by RT-qPCR revealed that rBCG-*Ag85A* induced systemic immune activation, characterized by increased *Il1B* and *Trl4* expression in splenocytes. In tumor tissues, rBCG-*Ag85A* significantly upregulated genes associated with apoptosis (*Bax*, *Bcl2*), oxidative stress (*Sod1*, *Cat*), inflammatory and immune signaling pathways (*Tlr4*, *Nfkb*, *Il12*, *Il1b*, *Casp1*), and modulation of pathways involved in cellular metabolism (*Mtor*) indicating enhanced modulation of the tumor microenvironment. Functionally, these molecular and immunological effects were associated with reduced tumor progression, slower tumor growth, and improved survival in B16F10 melanoma-bearing mice. Collectively, these findings demonstrate that rBCG-*Ag85A* promotes multifaceted antitumor activity through the modulation of apoptosis, inflammation and oxidative stress-related gene expression, highlighting its potential as a promising prophylactic vaccine strategy to prevent cutaneous malignant melanoma and supporting future studies aimed at elucidating the immune cell populations involved and optimizing combinatorial therapeutic approaches.

**Graphical abstract:**

**Figure:**
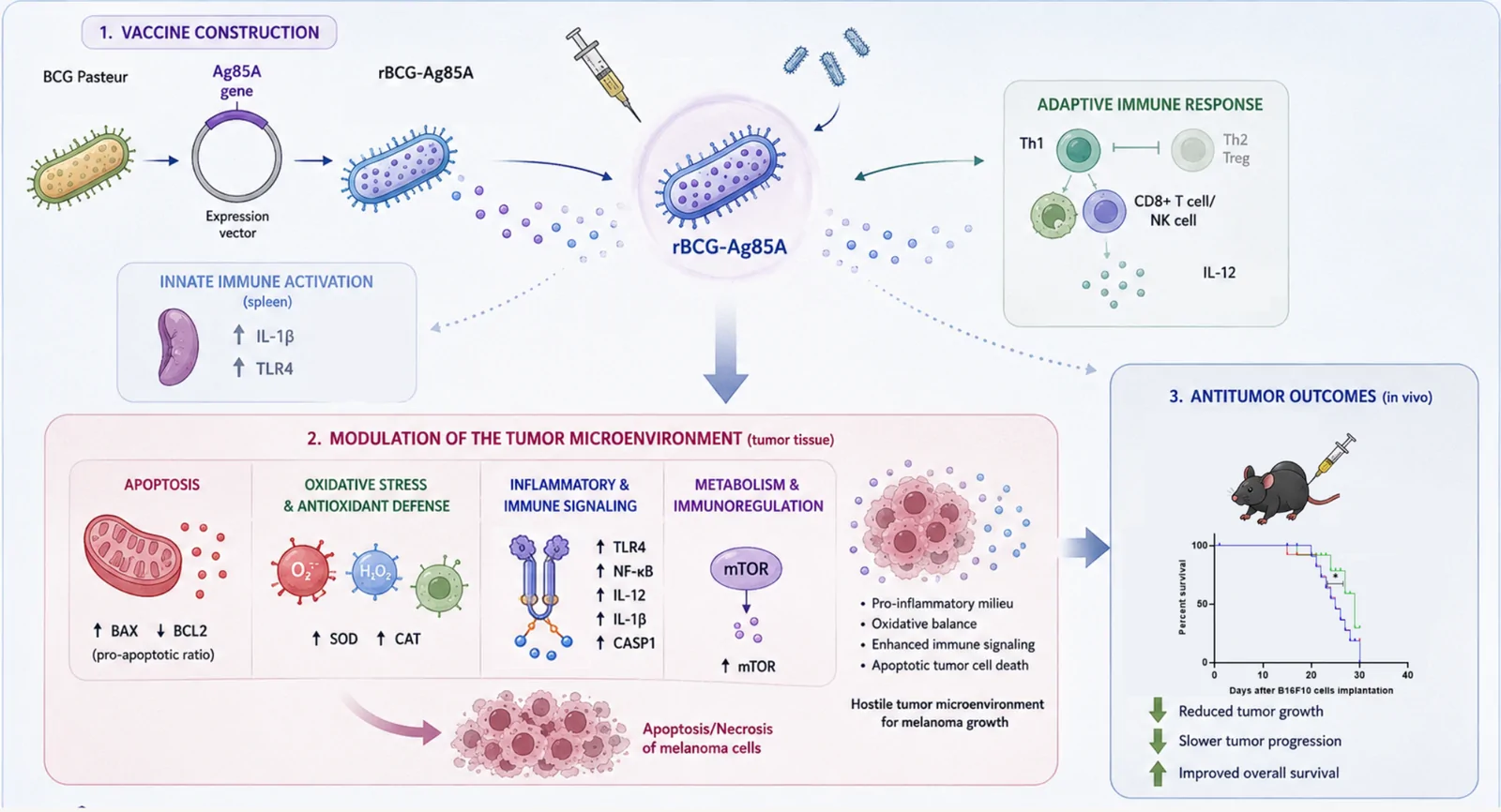
Antitumor effects of recombinant BCG-*Ag85A* in a melanoma model. rBCG-*Ag85A* enhanced systemic immune activation and modulated apoptosis, oxidative stress, inflammatory and immune-related pathways in tumor tissue, resulting in reduced tumor growth and improved survival in B16F10 melanoma-bearing mice. These findings highlight rBCG-Ag85A as a promising immunotherapeutic platform for cutaneous melanoma. Created in BioRender.

## Introduction

Cancer is a group of diseases characterized by uncontrolled cellular proliferation and the ability to invade surrounding tissues and distant organs. This dissemination process, known as metastasis, occurs through the bloodstream and/or lymphatic system and represents the primary cause of cancer-related mortality (World Health Organization 2017). Among the different cancer types, skin cancer is one of the most frequently diagnosed malignancies worldwide (Bray et al. 2024; Perez et al. 2022). These tumors are classified according to their cellular origin, mainly arising from the epidermis or dermis (Gershenwald et al. 2017; Fortarezza et al. 2024). Non-melanoma skin cancer, derived from epidermal cells, is the most prevalent form, whereas malignant melanoma originates from genetic alterations in melanocytes and is characterized by high aggressiveness and metastatic potential (Hasan et al. 2023).

Melanoma development is strongly associated with ultraviolet (UV) radiation exposure and hereditary factors, both involving distinct genetic mutations (Tímár and Ladányi 2022). Although melanoma accounts for only approximately 1% of all skin cancer cases, it is responsible for the majority of skin cancer-related deaths (Wang et al. 2023a ). Over the past five decades, its incidence and mortality rates have increased substantially (Raimondi et al. 2020), mainly due to its remarkable capacity for tissue invasion and systemic dissemination (Quail and Joyce 2013).

Despite significant advances in melanoma treatment, including immune checkpoint inhibitors and BRAF-targeted therapies, important challenges remain, particularly in advanced and metastatic disease (Shebrain et al. 2024). Historically, intralesional Bacillus Calmette-Guérin (BCG) was investigated as a therapeutic approach for melanoma (Morton et al. 1974; Benítez et al. 2019). The intrinsic adjuvant properties of BCG, together with its capacity to express heterologous antigens, support its continued investigation as a recombinant vaccine platform (Bastos et al. 2009; Qin et al. 2024).

BCG-based immunotherapy is known to induce robust immune activation through the recruitment and stimulation of multiple immune cell populations (Yang et al. 2017; Benítez et al. 2019). Although wild-type BCG alone is insufficient as a standalone melanoma therapy, its well-established safety profile and strong immunogenicity make it an attractive candidate for recombinant vaccine development. In this context, several genetically engineered BCG strains expressing heterologous antigens have demonstrated promising antitumor activity in preclinical models (Begnini et al. 2013; Mustafa et al. 2020), highlighting the potential of BCG as a platform for cancer immunotherapy.

Importantly, BCG remains the standard of care for non-muscle-invasive bladder cancer (Lidager et al. 2024), further supporting its potential application in other malignancies. Previous studies have explored recombinant BCG and auxotrophic ΔleuD strains for bladder cancer and metastatic melanoma, includinostg strains overexpressing endogenous *Mycobacterium bovis* antigens such as Antigen 85B (Ag85B) and Ag85A (Ag85B) (Begnini et al. 2013; Qin et al. 2024). These findings reinforce the feasibility of enhancing BCG-mediated antitumor responses through genetic engineering approaches.

Among the potential antigen candidates, Antigen 85A (Ag85A), a component of the antigen 85 complex produced by *Mycobacterium bovis*, stands out due to its high immunogenicity and ability to induce strong cellular immune responses. Ag85A has been shown to stimulate T-cell proliferation and interferon-gamma (IFN-γ) production in vaccinated individuals and experimental models, supporting its relevance in immunotherapy (Brennan et al. 2012; Wu et al. 2022). Furthermore, accumulating preclinical evidence suggests its potential application in cancer immunotherapy (Babaki et al. 2017; Tarrant et al. 2004; Zhang et al. 2012). Despite these advances, the development of effective recombinant BCG-based strategies targeting melanoma remains limited, particularly regarding prophylactic approaches and their capacity to induce protective antitumor immunity.

Therefore, this study aimed to generate novel recombinant BCG strains overexpressing Ag85A and to evaluate the prophylactic efficacy of rBCG-*Ag85A* vaccination against melanoma in both *in vitro* and *in vivo* models, as well as its capacity to modulate antitumor immune responses.

## Methodology

### Construction of plasmid pUS2000/*Ag85A*

#### Gene acquisition

The nucleotide sequence of the *Ag85A* gene of *Mycobacterium bovis*, was retrieved from the National Center for Biotechnology Information (NCBI: www.ncbi.nlm.nih.gov; accession Rv3804c). Gene design and sequence analysis were performed using Vector NTI 11 software (Invitrogen™, USA).

The gene was synthesized and cloned into the pUC19 cloning vector for propagation in *Escherichia coli*. To enable subcloning, restriction sites for the endonucleases *Xba*I and *Hind*III were added to the 5′ and 3′ ends of the sequence, respectively (GenOne Biotech©, Brazil).

These same restriction sites were also introduced into the expression vector pUS2000 (Dellagostin et al. 1993), which belong to the collection of the Vaccinology Laboratory at the Center for Technological Development—Biotechnology, Federal University of Pelotas (UFPel), for heterologous expression in *Mycobacterium bovis* BCG.

#### Strain and culture conditions

*Escherichia coli* TOP10 was grown in Luria–Bertani (LB) medium, supplemented with 1.5% agar for solid media, at 37 °C for 16 h. Kanamycin (50 µg/mL) was added when required for selection of recombinant colonies.

*Mycobacterium bovis* BCG Pasteur, and the recombinant strain rBCG-*Ag85A* were cultured in Middlebrook 7H9 broth (Difco, BD, Brazil) supplemented with 10% oleic acid-albumin-dextrose complex (OADC), 0.2% glycerol, and 0.05% Tween 80 at 37 °C for 7 days. For solid cultures, Middlebrook 7H10 agar supplemented with 10% OADC and 0.2% glycerol was used, with incubation at 37 °C for 21 days. Kanamycin (25 µg/mL) was added when necessary for selection of recombinant strain.

#### Cloning procedures

For cloning, 100 µL of electrocompetent cells were transformed with 50 ng of pUC19/*Ag85A* by electroporation (Gene Pulser, Bio-Rad), followed by plasmid extraction using a commercial miniprep kit. The *Ag85A* gene was excised from pUC19 via sequential digestion with *Xba*I and *Hind*III, purified from a 1% agarose gel, and ligated into similarly digested pUS2000 expression vector using T4 DNA ligase.

The resulting construct (pUS2000/*Ag85A*) was transformed into *E. coli* TOP10, and a recombinant clone was selected on LB agar containing kanamycin. Positive clone were screened by agarose gel electrophoresis from a 1% agarose gel following plasmid extraction or rapid lysis, and confirmed by restriction enzyme digestion, demonstrating successful insertion and release of the *Ag85A* gene.

#### Transformation of *Mycobacterium bovis* BCG Pasteur

*Mycobacterium bovis* BCG Pasteur was cultured in 7H9 medium supplemented with OADC and Tween 80 at 37 °C for approximately 5 days until the desired optical density was reached. Cells were harvested by centrifugation (14.000 rpm, 15 min), washed with pyrogen-free water, and sequentially resuspended in 10% glycerol to obtain electrocompetent cells.

For transformation, 50 µg of pUS2000/*Ag85A* plasmid was added to 100 µL of electrocompetent cells and incubated on ice for 10 min. Electroporation was performed using a Gene Pulser (Bio-Rad) under the following conditions: 2.5 kV, 25 µF, and 800 Ω. Immediately after, 500 µL of supplemented 7H9 medium was added, and cells were incubated at 37 °C under agitation. The cultures were then plated on 7H10 agar supplemented with OADC and kanamycin, followed by incubation at 37 °C for approximately 21 days. The resulting recombinant strain was designated rBCG-*Ag85A*.

#### Characterization of gene transcription by real-time PCR (RT-qPCR)

Gene transcription was evaluated by RT-qPCR. Briefly, 10 mL cultures of BCG Pasteur and rBCG-*Ag85A* were centrifuged (4,000 × g, 15 min), and cell pellets were lysed using a ribolyser (Hybaid, UK) for four cycles of 45 s. Total RNA was extracted using TRIzol® reagent (Invitrogen™, USA), and RNA concentration and purity were determined with a NanoVue™ Plus spectrophotometer (GE Healthcare Life Sciences, USA). Only samples with OD₂₆₀/₂₈₀ ≥ 2.0 were used.

cDNA synthesis was performed from 1 µg of total RNA using the High-Capacity cDNA Reverse Transcription Kit (Applied Biosystems, UK), according to the manufacturer’s instructions. qPCR reactions were carried out on a Stratagene Mx3005P Real-Time PCR System (Agilent Technologies, USA) using SYBR Green PCR Master Mix (Applied Biosystems, UK) and gene-specific primers (*Ag85A* forward: 5’CAGGCTCGTCGGCAC and reverse: 5’GGGCCCCACATGTCC). All reactions were performed in triplicate, and relative expression levels were calculated using the 2^ − ΔΔCt method, using *leu*D as the endogenous reference gene (forward: 5′-TCTTTCTGAAGCGGGTCACC-3′ and reverse: 5′-GCTTCCACAGGAGTTCCACA-3′). The relative expression levels were statistically compared between the BCG Pasteur and rBCG-*Ag85A* strains using Student’s *t*-test, and differences were considered statistically significant when *p* < 0.05.

#### Immunization of mice

A total of 48 female C57BL/6 mice (23 ± 2 g), aged seven to eight weeks, were obtained from the Federal University of Pelotas (UFPel). Animals were housed under specific pathogen-free (SPF) conditions at 22 °C with a 12-h light/dark cycle in the UFPel animal facility.

**Fig. 1:**
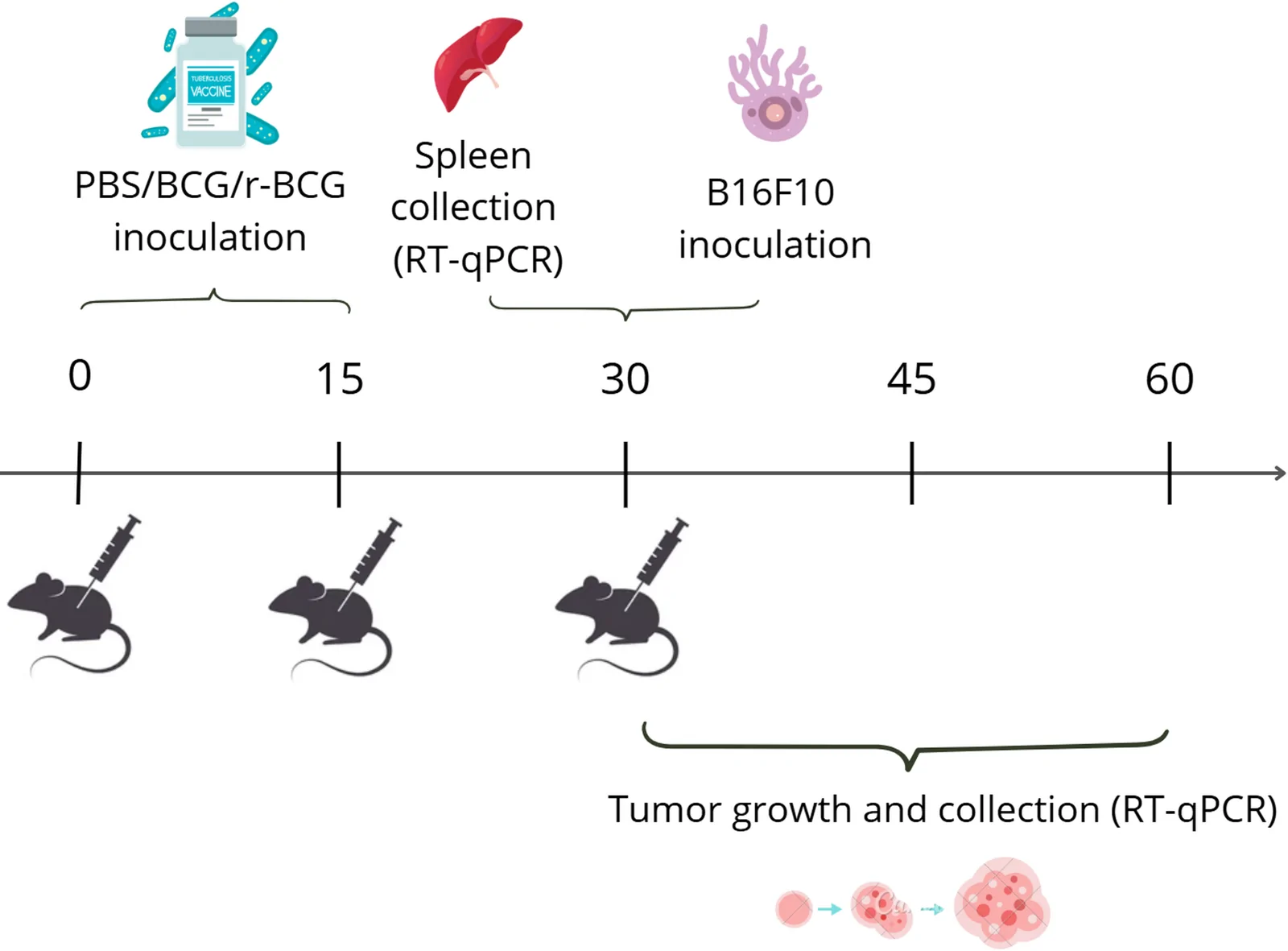
Experimental design of the immunization and tumor challenge protocol. Mice were immunized with rBCG on days 0 and 15. Fifteen days after the second immunization, six animals per group were euthanized for spleen collection for the immunological analyses (RT-qPCR). On the 30th day after the first rBCG inoculation, the remaining ten animals were subcutaneously inoculated with tumor cells, as illustrated in the schematic. Tumor growth and survival were monitored for 30 days following the tumor challenge. Tumor collection for the immunological analyses (RT-qPCR). Schematic illustration created with Canva (Canva, Australia).

After a 7-day acclimatization period, mice were randomly assigned to three experimental groups (n = 16 per group). Each animal received two intraperitoneal inoculations of PBS, BCG, or rBCG at 15-day intervals. On day 30 after the first inoculation, tumor cells were administered via the dorsal subcutaneous route. Tumor volume and survival rate were evaluated for 30 days following tumor cell inoculation, as illustrated in the experimental design (Fig. 1). All experimental procedures involving animals were approved by the Animal Ethics Committee of UFPel (CEUA approval no. 021711/2019–94).

#### Tumor inoculation

Murine melanoma B16F10 cells were obtained from the Rio de Janeiro Cell Bank (BCRJ). Cells were maintained under standard culture conditions (37 °C, 5% CO₂) and harvested from culture flasks using trypsin (Sigma, USA), followed by three washes with PBS. On the day of inoculation, a suspension containing 1 × 10^5^ B16F10 melanoma cells in 200 μL of PBS was prepared for administration.

#### Treatment scheme

Mice in Group 1 received an intraperitoneal injection of 100 μL of PBS. Group 2 received an intraperitoneal injection of 1 × 10⁶ CFU of *Mycobacterium bovis* BCG Pasteur suspended in 100 μL of PBS. Group 3 received an intraperitoneal injection of 1 × 10⁶ CFU of recombinant rBCG-*Ag85A* suspended in 100 μL of PBS. BCG liquid culture was used in the vaccine preparation.

Fifteen days after the second inoculation, six animals per group were euthanized for spleen collection. The remaining ten animals in each group were subcutaneously inoculated in the dorsal region with 1 × 10^5^ murine B16F10 melanoma cells suspended in 200 μL of PBS, as illustrated in the vaccination scheme (Table 1).

**Table 1:** Vaccination schedule for the different experimental groups

Experimental Groups	1st inoculation (day 0) n = 16	2nd inoculation (day 15) n = 16	3rd inoculation (day 30) n = 10
Group 1	PBS	PBS	B16F10
Group 2	BCG Pasteur	BCG Pasteur	B16F10
Group 3	rBCG-*Ag85A*	rBCG-*Ag85A*	B16F10

PBS: Phosphate-buffered saline. n: Number of mice per group. BCG Pasteur: Wild-type BCG strain. rBCG-*Ag85A*: Recombinant BCG vaccine overexpressing the *Ag85A* gene inserted into a recombinant plasmid. B16F10: Melanoma murine.

#### Spleen collection and cytokine assessment

Spleens were aseptically collected from immunized mice (n = 6 per group) 15 days after the last rBCG vaccination to evaluate the production of cytokines. Animals were euthanized, and the skin was disinfected with 70% ethanol prior to spleen removal using sterile scissors under aseptic conditions.

Collected spleens were washed in PBS in Petri dishes and mechanically dissociated using a 5 mL syringe plunger through a 70 μm cell strainer to obtain a single-cell suspension. After centrifugation, the cell pellet was resuspended in the red blood cell lysis buffer. Splenocytes were isolated by centrifugation and subsequently cultured in 12-well plates.

Splenocytes isolated from all experimental groups (PBS, BCG Pasteur, and rBCG-Ag85A) were seeded in 12-well plates to evaluate antigen-specific systemic immune responses. Cells were individually restimulated with either 1 × 10⁶ CFU of BCG Pasteur, 1 × 10⁶ CFU of rBCG-Ag85A, 8 ng/mL concanavalin A (positive control), or 0.9% saline solution (negative control). The cultures were incubated for 48 h under standard culture conditions (37 °C, 5% CO₂). Following this incubation period, cells were collected for total RNA extraction using TRIzol® reagent (Invitrogen).

#### Immunological profile in the tumor microenvironment

Total messenger RNA (mRNA) was extracted from collected B16F10 tumors using a TRIzol-based protocol involving chloroform, isopropanol, ethanol, and RNase-free water. cDNA synthesis was performed using oligo(dT)12–18 primers and reverse transcriptase, according to the manufacturer’s instructions for the High-Capacity cDNA Reverse Transcription Kit. Real-time PCR (qPCR) was performed using primers described in Table 2. Gene expression analysis was performed in tumors treated with PBS, BCG, or rBCG. Expression levels of genes associated with apoptosis, oxidative stress markers, inflammatory, immune response, and cell signaling pathway were evaluated.

**Table 2:** Murine gene-specific primer sequences used for real-time quantitative PCR (RT-qPCR) analysis

Gene	Sequence 5’-3’
*Gapdh*	F: GGGTGAGGCCGGTGCTGAG
	R:TGGGGGTAGGAACACGGAAGG
*Bcl2*	F: TGGGATGCCTTTGTGGAACT
	R: GAGACAGCCAGGAGAAATCA
*Bax*	F: CCACCAGCTCTGAACAGATC
	R: CAGCTTCTTGGTGGACGCAT
*Casp1*	F: CGTCTTGCCCTCATTATCTG
	R: TCACCTCTTTCACCATCTCC
*Cat*	F:GAACGAGGAGGAGAGGAAAC
	R: TGAAATTCTTGACCGCTTTC
*Sod1*	F: TGGTGGTCCATGAGA
	R: GTTTACTGCGCAATC
*Mtor*	F: AGAAGGGTCTCCAAGGACGACT
	R: GCAGGACACAAAGGCAGCATTG
*Tlr4*	F: GCCTTTCAGGGAATTAAGCTCC
	R: AGATCAACCGATGGACGTGTAA
*Nfkb*	F: GCTTTCGCAGGAGCATTAAC
	R: CCGAAGCAGGAGCTATCAAC
*Il12*	F: AGCACCAGCTTCTTCATCAGG
	R: CCTTTCTGGTTACACCCCTCC
*Il1b*	F: GCTGAAAGCTCTCCACCTCAATG
	R: CAGCTCATATGGGTCCGACA

*Gapdh*, glyceraldehyde-3-phosphate dehydrogenase; *Bcl2*, B-cell lymphoma 2; *Bax*, BCL2-associated X protein; *Casp1*, caspase 1; *Cat*, catalase; *Sod1*, superoxide dismutase 1; *Mtor*, mechanistic target of rapamycin kinase; *Tlr4*, toll-like receptor 4; *Nfkb*, nuclear factor kappa B subunit 1; *Il12*, interleukin 12; *Il1b*, interleukin 1 beta.

Total mRNA was extracted using TRIzol reagent (Invitrogen™, USA) and quantified with a NanoVue Plus spectrophotometer (GE^®^). cDNA synthesis was performed using the High-Capacity cDNA Reverse Transcription Kit (Applied Biosystems™) according to the manufacturer’s protocol. Real-time PCR reactions were conducted using SYBR Green PCR Master Mix in a Stratagene Mx3005P Real-Time PCR System (Agilent Technologies, USA). Gene expression levels in the groups treated with BCG Pasteur and rBCG-*Ag85A* were compared to the control group (PBS). All reactions were performed in triplicate, and relative expression levels were calculated using the 2^ − ΔΔCt method, with *Gapdh* as the endogenous reference gene (Livak and Schmittgen 2001).

### Tumor volume measurement

Ten mice per group were used for tumor growth and survival analyses, while the remaining animals were used for ex vivo assays. Following subcutaneous inoculation of B16F10 cells, mice were monitored every two days for visible tumor development. Tumor dimensions were measured using a digital caliper. The largest longitudinal diameter (D) and the largest transverse diameter (d) were recorded. Tumor volume (mm^3^) was calculated using the formula: Tumor volume = D × d^2 × 0.52. Tumor growth was monitored for up to three weeks, and measurements from the rBCG-treated group was compared with the untreated B16F10-challenged control group. Tumor volume was analyzed using two-way ANOVA.

### *In vivo* survival analysis

After subcutaneous inoculation of B16F10 cells, animals were monitored every two days. Clinical assessments and tumor measurements were performed by an independent investigator blinded to group allocation. Animals were euthanized upon presentation of clinical signs of distress, including anorexia, cachexia, marked tumor enlargement, impaired mobility, tumors exceeding 2000 mm^3^, or ulceration. Survival was recorded for 30 days following tumor inoculation. Kaplan–Meier survival curves were generated, and differences between groups were analyzed using the log-rank test.

### Statistical analysis

Statistical analyses of relative gene expression were conducted using relative gene expression calculated using the 2^−ΔΔCt^ method, applying Student’s *t*-test and one-way ANOVA followed by Tukey’s post hoc test. Tumor volume was analyzed using two-way ANOVA. Survival analysis using the Kaplan–Meier method with the logrank test. Data are presented as mean ± SEM. Statistical significance was defined as p < 0.05 (*p < 0.05; **p < 0.001; ***p < 0.001; ****p < 0,0001; ns, not significant). All statistical analyses were performed using GraphPad Prism 8 software.

## Results

### Characterization of *Ag85A* in BCG by RT-qPCR

**Fig. 2:**
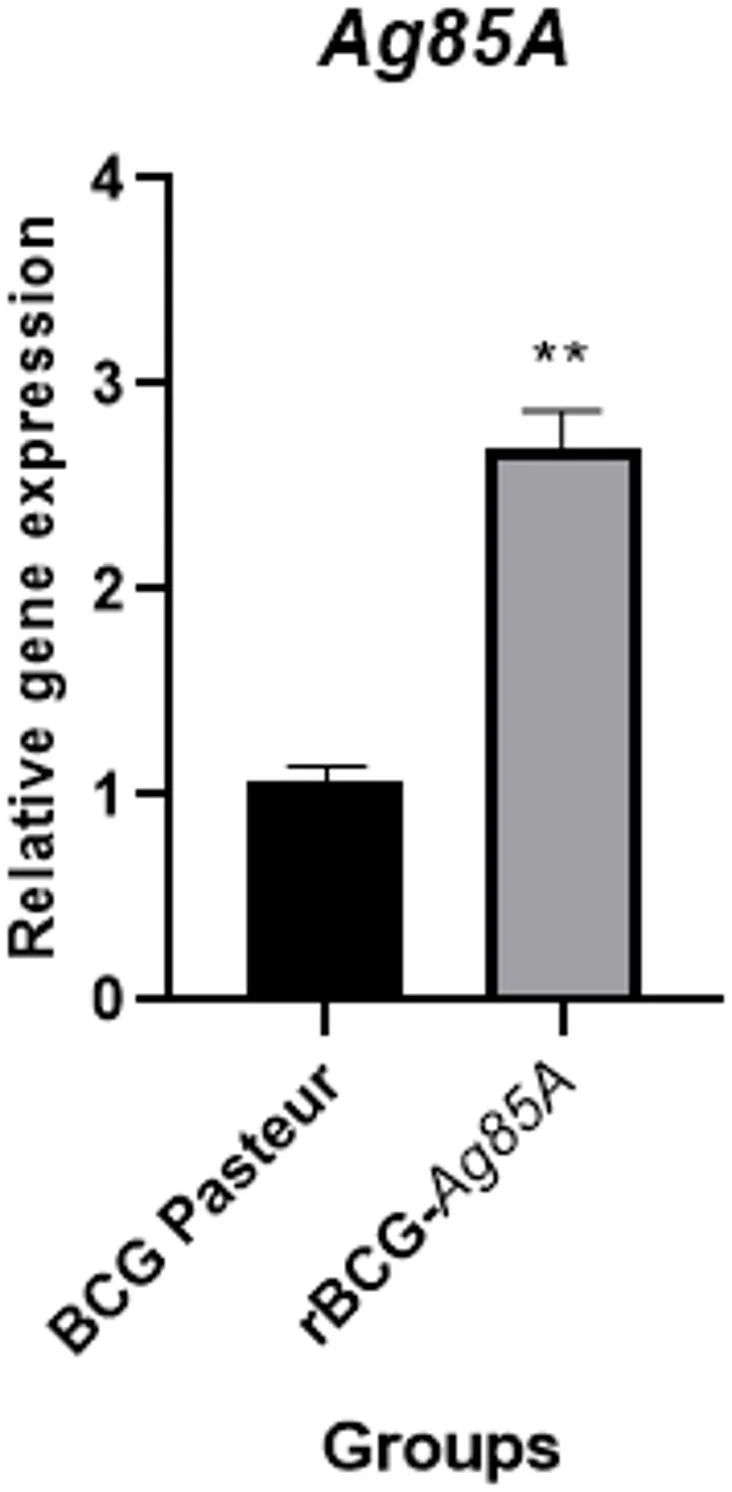
Relative gene expression analysis by RT-qPCR demonstrated increased *Ag85A* expression in the rBCG-*Ag85A* strain compared with the parental BCG Pasteur strain (control). Data are presented as the mean ± standard error of the mean (SEM). Statistical analysis using Student’s *t*-test in GraphPad Prism 8 software. (*) indicates significant differences compared to the control group. *p < 0.05, **p < 0.01

Results of the characterization of *Ag85A* by RT-qPCR show that rBCG at levels approximately three times higher than those observed in the parental BCG Pasteur strain (control) (Fig. 2). These findings indicate earlier amplification and suggest successful overexpression of the target gene in the recombinant BCG.

### Cytokine profiling in the spleen cells

**Fig. 3:**
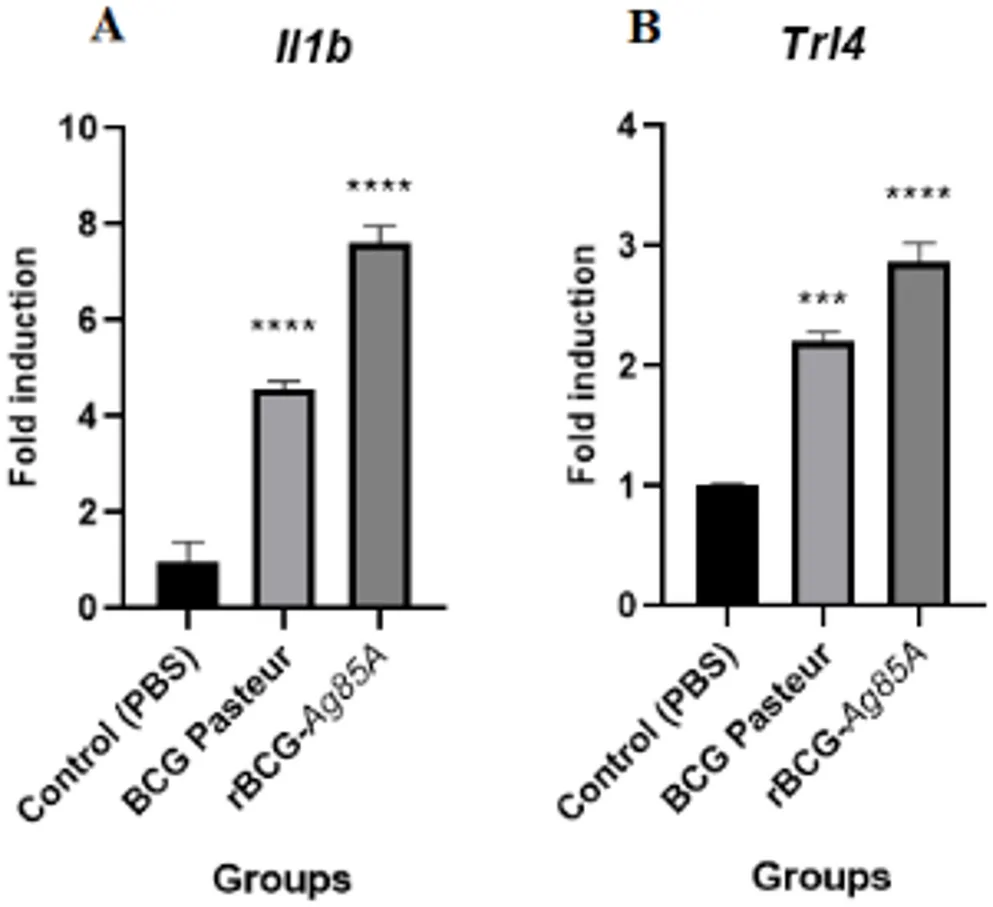
Cytokine production by the immunized mice splenocytes with and without stimulation with the rBCG vaccine *in vitro*. Relative gene expression of (A) *Il1b* and (B) *Tlr4* in spleen samples from treated and control groups, determined by real-time PCR. Data are presented as the mean ± standard error of the mean (SEM). Statistical analysis was performed using one-way ANOVA followed by Tukey’s post hoc test in GraphPad Prism 8 software. (*) indicates significant differences compared to the control group. *p < 0.05, **p < 0.01, ***p < 0.001, ****p < 0.0001

The results of cytokine profiling in spleen cells demonstrate increased relative gene expression of *Il1b* (Fig. 3A) and *Tlr4* (Fig. 3B) in the rBCG-*Ag85A* treated group compared to the control group (PBS). Additionally, a reduction in *Il1b* expression was observed in the group treated with wild-type BCG (BCG Pasteur) when compared to the control group (Fig. 3A).

### Immunological profile in the tumor microenvironment

#### Apoptosis-related genes

**Fig. 4:**
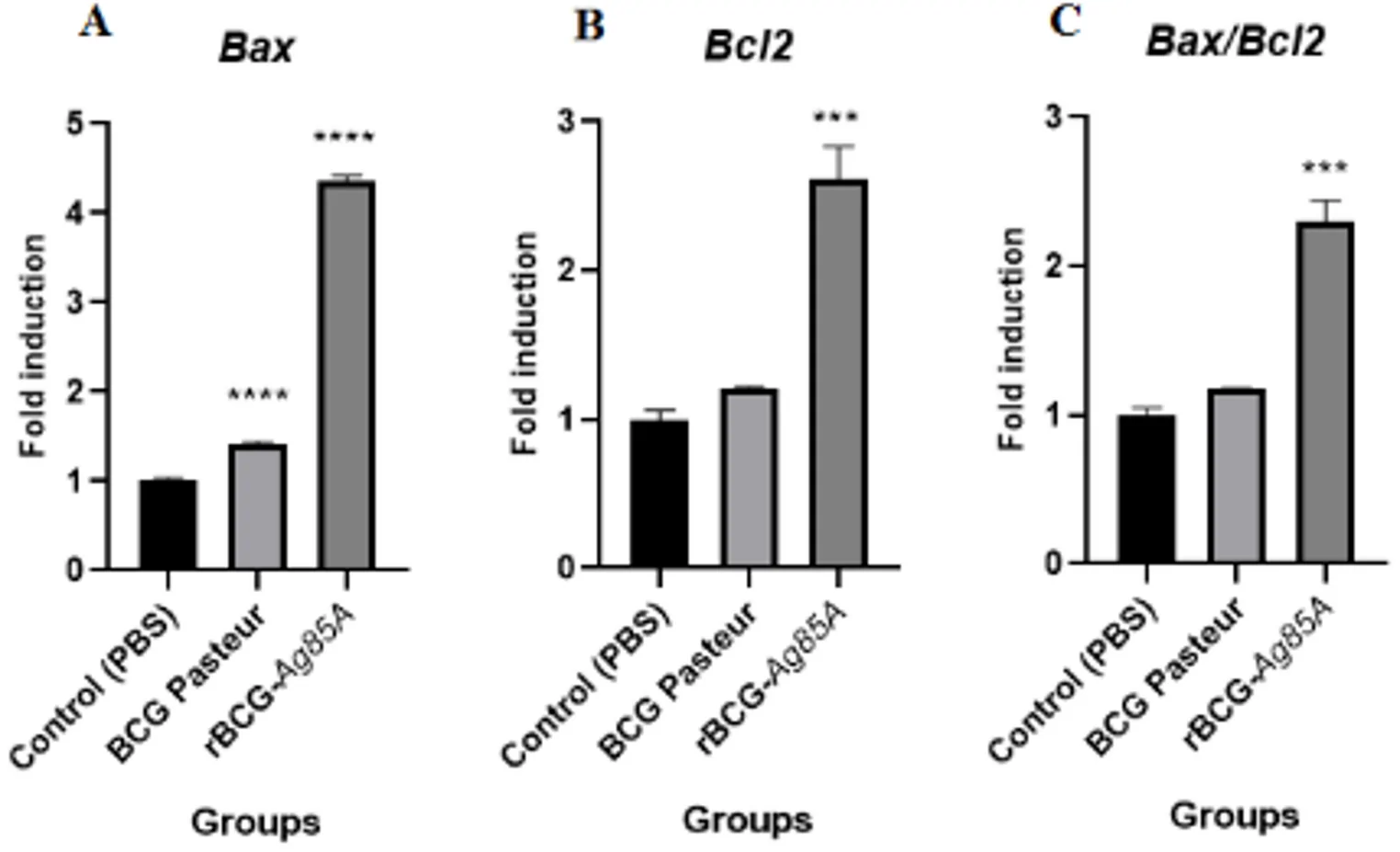
Relative gene expression of (**A**) *Bax* and (**B**) *Bcl2* in tumor samples from treated rBCG, BCG Pasteur and control groups, determined by real-time PCR. (**C**) Analysis of the Bax/Bcl2 ratio. Data are presented as mean ± SEM. Statistical analysis was performed using one-way ANOVA followed by Tukey’s post hoc test in GraphPad Prism 8 software. (*) indicates significant differences compared to the control group. *p < 0.05, **p < 0.01, ***p < 0.001****p < 0.0001

In tumor samples, a significant increase in the relative gene expression of *Bax* (Fig. 4A) and *Bcl2* (Fig. 4B) was observed in the groups treated with rBCG compared to the control group. In addition, a significant upregulation of *BAX* expression was also detected in the group treated with BCG Pasteur compared to the control group (Fig. 4A). Also Analysis of the balance between pro-apoptotic (*Bax*) and anti-apoptotic (*Bcl2*) proteins was determined by calculating the *Bax/Bcl2* ratio. Higher values indicate apoptosis induction, while lower values are associated with inhibition of cell death. A ratio greater than 1 was observed in the rBCG-*Ag85A* treated group, indicating a shift toward a pro-apoptotic profile (Fig. 4C).

#### Oxidative stress markers

**Fig. 5:**
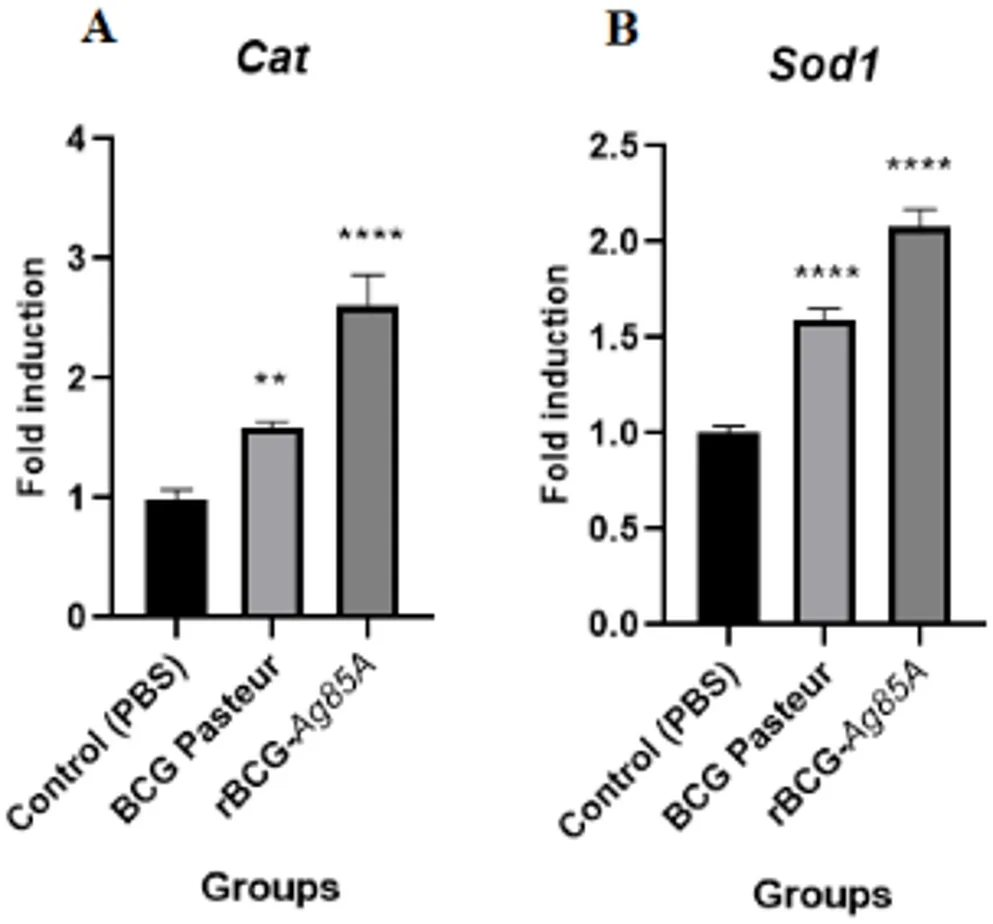
Relative expression of oxidative stress-related genes: (**A**) *Cat* and (**B**) *Sod1* in tumor samples from treated rBCG, BCG Pasteur and control groups, assessed by real-time PCR. Results are expressed as mean ± SEM. Statistical significance was determined using one-way ANOVA followed by Tukey’s post hoc test in GraphPad Prism 8 software. (*) indicates significant differences compared to the control group. *p < 0.05, **p < 0.01, ***p < 0.001****p < 0.0001

A significant increase in the relative expression of *Cat* (Fig. 5A) and *Sod1* (Fig. 5B) was observed in the groups treated with rBCG and BCG Pasteur compared to the control group.

#### Inflammatory and immune response

**Fig. 6:**
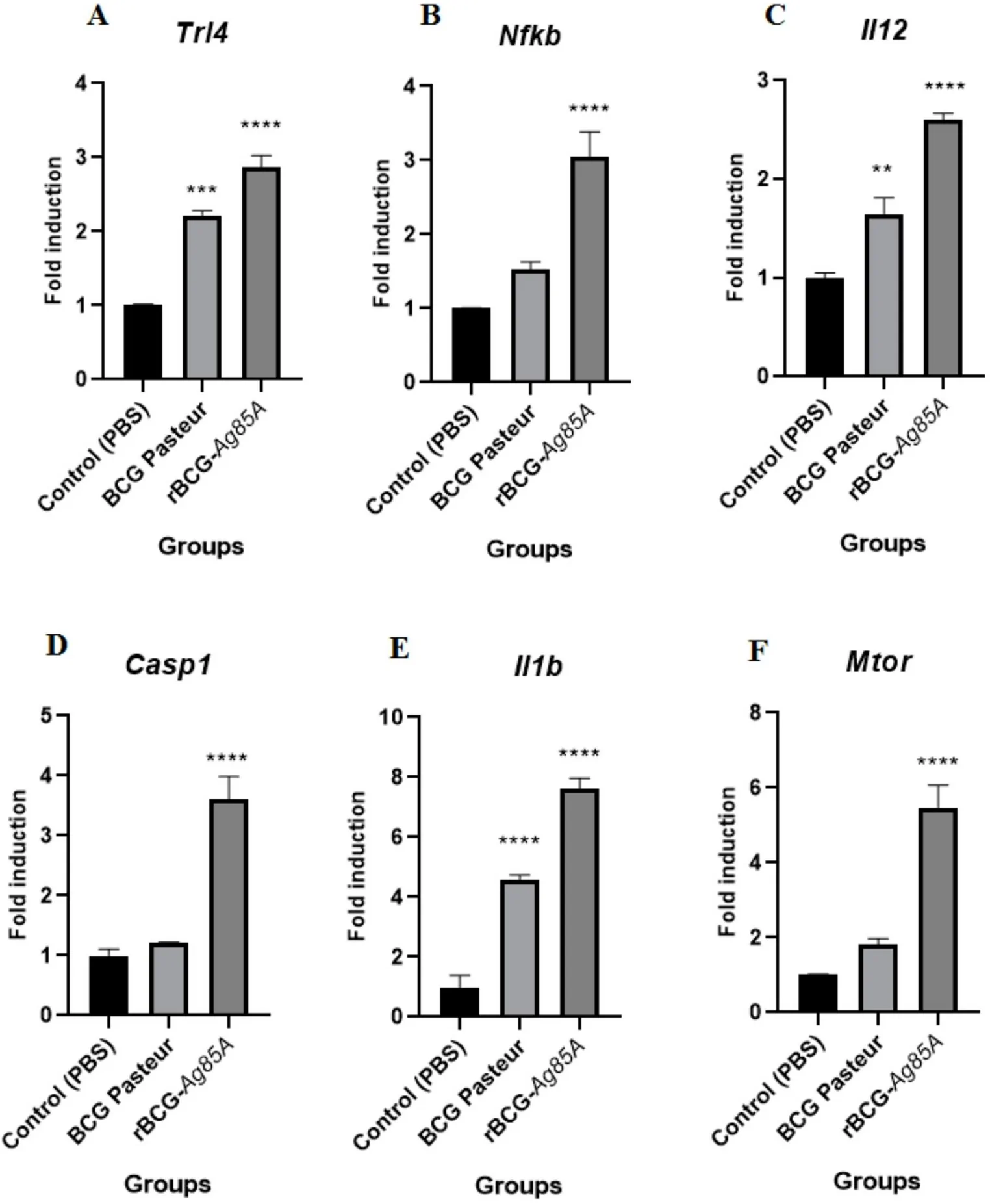
Relative gene expression of inflammatory, immune response and cell signaling pathway markers: (**A**) *Tlr4*, (**B**) *Nfkb*, (**C**) *Il12*, (**D**) *Casp1*, (**E**) *Il1b* and (**F**) *Mtor* in tumor samples from treated with rBCG, BCG Pasteur and control groups, evaluated by real-time PCR. Data are presented as mean ± SEM. Statistical analysis was performed using one-way ANOVA followed by Tukey’s post hoc test in GraphPad Prism 8 software. (*) indicates significant differences compared to the control group. *p < 0.05, **p < 0.01, ***p < 0.001****p < 0.0001

In tumor tissue, a statistically significant increase in the relative expression of *Tlr4*, *Nfkb, Il12, Casp1, Il1b* and *Mtor* (Fig. 6A–F) was observed in the groups treated with rBCG-*Ag85A* compared to the control group. Additionally, an increase in the expression of *Tlr4, Il12,* and *Il1b* was also observed in the group treated with BCG Pasteur compared to the control group (Fig. 6A, C and E).

### Tumor volume measurement and survival analysis

**Fig. 7:**
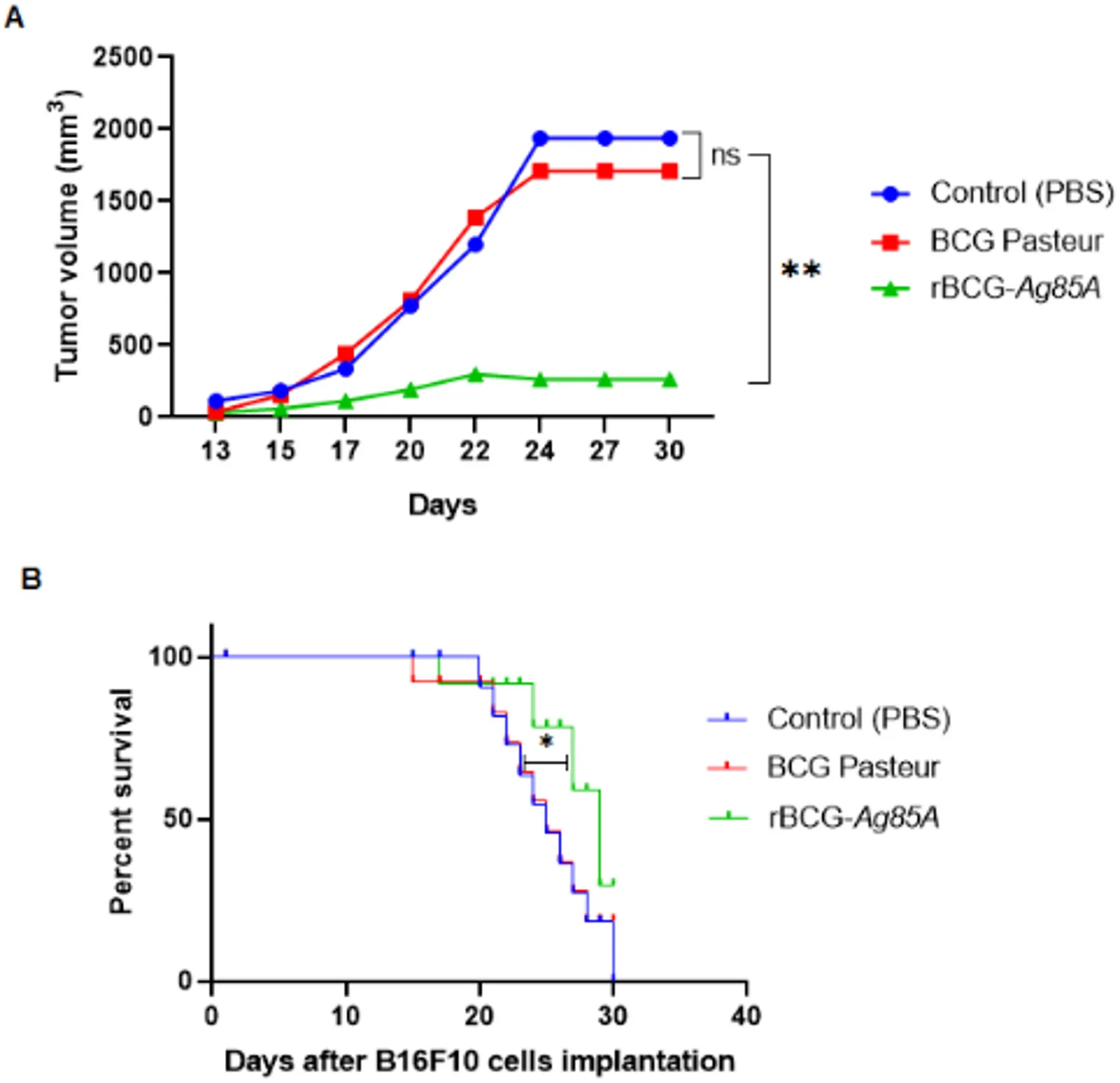
Melanoma tumor volume progression over time. (**A**) Tumor volume (mm^3^) was measured in mice treated with rBCG, BCG Pasteur and compared to the control group at different time points. Statistical analysis was performed using the two-way ANOVA in GraphPad Prism 8 software. (**B**) Survival curves of mice following B16F10 inoculation. Mice treated with rBCG showed improved survival compared to the control group, indicating enhanced antitumor efficacy. Statistical analysis was performed using the Kaplan–Meier method with the logrank test in GraphPad Prism 8 software. (*) indicates significant differences compared to the control group. *p < 0.05

Tumor volumes were monitored over time in groups treated with BCG Pasteur and rBCG, compared to the control group. Mice in the control group showed rapid, uncontrolled tumor progression, reaching an average volume of 1939.50 mm^3^ by day 30. In contrast, animals treated with rBCG-*Ag85A* exhibited a highly significant restriction in tumor growth, maintaining a mean tumor volume of 249.43 mm^3^ at the same timepoint (p < 0.05; Fig. 7A). This represents approximately 87.15% reduction in tumor volume compared to the control group on day 30. The parental BCG Pasteur strain also slowed tumor growth (1709.24 mm^3^), but its efficacy was significantly less pronounced than that of the recombinant strain over the entire observation period (Fig. 7A).

Survival curves following B16F10 melanoma cell inoculation reflected the tumor growth kinetics, demonstrating enhanced antitumor efficacy in the rBCG-*Ag85A* group. The median survival time of the control group was 17 days, with 100% mortality reached by day 30. Treatment with parental *BCG* Pasteur resulted in a median survival of 15 days. Remarkably, mice immunized with rBCG-*Ag85A* exhibited the greatest therapeutic benefit, with median survival not reached within the 30-day monitoring period (log-rank test, *p* < 0.05 vs. control; Fig. 7B), and 30% of the animals surviving until the end of the experimental period.

## Discussion

Melanoma is considered the most immunogenic solid tumor and, consequently, has served as a primary model for the development of tumor vaccines, both in preclinical and clinical settings. Advances in melanoma vaccination strategies have largely stemmed from a deeper understanding of the cellular interactions required to elicit a tumor-specific immune response (Brinckerhoff et al. 2000; Seretis et al. 2025). BCG is a promising live vector for multivalent vaccines and antitumor therapies (Borges et al. 2024; Kremenovic and Schenk 2020; Singh et al. 2021). Genetic modifications have enhanced its effects in bladder cancer models, both *in vitro* and *in vivo*. For example, a BCG strain overexpressing *Ag85B* from *Mycobacterium bovis* increased cytotoxicity against bladder cancer cells (Begnini et al. 2013). Furthermore, the mixed dendritic cell vaccine combined with Ag85A exhibited a marked inhibitory effect on tumor progression in mice, reinforcing the antitumor potential of Ag85A-based immunotherapeutic strategies (Zhai et al. 2018). This combination of apoptosis and immune activation demonstrates enhanced antitumor potential in response to vaccination using BCG.

Ag85A, a 31 kDa protein, forms part of the *fbpA* complex along with Ag85B and Ag85C (Babaki et al. 2017). This complex, present in both *Mycobacterium tuberculosis* and *Mycobacterium bovis* BCG, is a major component of bacterial culture filtrates and is closely associated with the cell wall (Wiker and Harboe 1992). The three proteins are encoded by distinct genes—*fbpA*, *fbpB*, and *fbpC2*, located at Rv3804c, Rv1886c, and Rv0129c, respectively (Wiker and Harboe 1992). The Ag85 complex plays a crucial role in mycobacterial virulence, supporting survival within macrophages while also promoting host immune responses. Its mycolyl-transferase activity facilitates the attachment of mycolic acids to arabinogalactan and contributes to cord factor biogenesis, which is essential for cell wall integrity (Kuo et al. 2012). Epitope mapping has further shown that different peptides from the Ag85 complex can effectively stimulate human T cells (Huygen 2014; Yuk and Jo 2014). This study aimed to generate recombinant BCG strain overexpressing *Ag85A* by cloning the *Ag85A* gene into an expression vector and to evaluate the prophylactic efficacy of rBCG-*Ag85A* vaccination against melanoma, as well as its ability to induce tumor-targeted immune responses*.*

Cytokine profiling in spleen cells revealed that treatment with rBCG led to a significant increase in the relative expression of *Il1b* and *Tlr4*, while BCG Pasteur also modulated *Il1b* expression compared to controls (Fig. 3). These findings suggest that rBCG-Ag85A is capable of initiating an early innate immune signaling cascade systemically, which may contribute to priming antitumor responses observed at the tumor site. Although the evaluation of splenocytes in this study was limited to these two key markers of innate activation and did not encompass a full characterization of systemic Th1 cytokines, such as *Ifn-γ* and *Tnf-α*, the upregulation of *Tlr4* and *Il1b* provides a preliminary indication of systemic responsiveness. These initial pathways are consistent with previous studies showing that heterologous BCG administration in a prime-boost regimen with a DNA vaccine expressing Ag85A enhances protective efficacy against *Mycobacterium tuberculosis* in mice (Dou et al. 2010; Wu et al. 2022). Future investigations evaluating a broader panel of systemic lymphoid cytokines will be essential to fully elucidate the polarization of the adaptive systemic immune response induced by this recombinant strain.

In tumor tissue, rBCG-*Ag85A* treatment significantly upregulated genes associated with apoptosis (*Bax, Bcl2*) (Fig. 4), and oxidative stress (*Sod1, Cat*) (Fig. 5) inflammation and immune response (*Tlr4, Nfkb, Il12, Il1b, Casp1*) (Fig. 6) compared to controls. These molecular changes in the tumor microenvironment suggest that rBCG promotes apoptosis and stimulates immune-mediated tumor suppression more effectively than BCG Pasteur. In parallel, these effects were consistent with previous studies in tuberculosis, in which rBCG elicited enhanced inflammatory responses, where evaluation of early immune responses showed that rBCG triggered significantly stronger pro-inflammatory responses than the parental BCG, characterized by elevated levels of *TNF-α, IL-17*, and *IFN-γ*, which led to enhanced activation of CD4⁺ and CD8⁺ T cells (De Almeida et al. 2026). Together, these findings demonstrate that rBCG exerts a multifaceted antitumor effect by simultaneously modulating apoptotic pathways, inflammatory signaling, oxidative stress, and T cell-mediated immunity.

The tumor microenvironment plays a central role in shaping the host immune response, influencing the balance between effective antitumor immunity and tumor-induced immune suppression. Both local and systemic factors within tumors create a profoundly immunosuppressive milieu, which represents a major obstacle for cancer therapy (Tartey et al. 2021; Tufail et al. 2025). Effective immunotherapies aim to overcome this suppression, reprogramming immune, stromal, and tumor cells to restore robust antitumor responses (Liu et al. 2024). In line with this, studies in tuberculosis have shown that human M2 macrophages infected with rBCG upregulate inflammatory genes (*TAP1, GBP1, SLAMF7, TNIP1, IL-6*) and secrete pro-inflammatory cytokines and tissue-repair factors, including *MCP-3* and EGF (Dos Santos et al. 2022). These results highlight the capacity of rBCG to counteract immunosuppressive mechanisms, enhancing immune cell activation and potentially improving control of both tumor cells and pathogens.

The tumor microenvironment plays a central role in shaping the host immune response, influencing the balance between effective antitumor immunity and tumor-induced immune suppression. Both local and systemic factors within tumors create a profoundly immunosuppressive milieu, which represents a major obstacle for cancer therapy (Tartey et al. 2021; Tufail et al. 2025). Effective immunotherapies aim to overcome this suppression, reprogramming immune, stromal, and tumor cells to restore robust antitumor responses (Liu et al. 2024). In line with this, studies in tuberculosis have shown that human M2 macrophages infected with rBCG upregulate inflammatory genes (*TAP1, GBP1, SLAMF7, TNIP1, IL-6*) and secrete pro-inflammatory cytokines and tissue-repair factors, including *MCP-3* and EGF (Dos Santos et al. 2022). These results highlight the capacity of rBCG to counteract immunosuppressive mechanisms, enhancing immune cell activation and potentially improving control of both tumor cells and pathogens. It is important to recognize, however, that tumor-associated macrophages (TAMs) comprise a highly heterogeneous population whose functional states extend beyond the traditional M1/M2 polarization paradigm. Recent evidence indicates that TAM phenotype, function, and spatial distribution are dynamically shaped by the tumor microenvironment, giving rise to distinct macrophage subsets that differentially regulate tumor progression, angiogenesis, immune suppression, and therapeutic responses (Grayson et al. 2024). Moreover, specific TAM subsets have been directly implicated in resistance to cancer immunotherapy. In hepatocellular carcinoma, CX3CR1⁺ TAMs were shown to promote T-cell exhaustion through IL-27 secretion following anti-PD1 treatment, thereby limiting the efficacy of immune checkpoint blockade. Notably, pharmacological targeting of this macrophage subset enhanced the therapeutic response to anti-PD1 therapy, highlighting the functional diversity of TAMs and their potential as therapeutic targets (Xiang et al. 2024). Collectively, these findings reinforce that macrophage populations should be viewed as a spectrum of specialized phenotypes rather than a binary M1/M2 classification, emphasizing the importance of comprehensive immune-cell characterization in studies investigating antitumor immunotherapies.

The molecular effects observed in this study were functionally reflected in a robust and quantitative reduction in tumor progression together with improved survival. Specifically, the rBCG-*Ag85A*-treated group demonstrated an 87.15% reduction in mean tumor volume by day 30 (249.43 mm^3^) compared to untreated controls (1939.50 mm^3^; Fig. 7A), significantly outperforming the conventional *BCG* Pasteur strain. This marked suppression of tumor growth translated into a clear survival advantage, with the median survival increasing from 17 days in the control group to not reached within the 30-day monitoring period in the rBCG-*Ag85A* cohort, and 30% of the animals surviving until the end of the observation period (Fig. 7B). Additionally, the upregulation of *Mtor* observed in tumor tissues from the rBCG-*Ag85A* treated group suggests the involvement of signaling pathways associated with cellular metabolism and immune response regulation (Fig. 6F). This finding is consistent with the increased *Tlr4* expression observed in both splenocytes and tumor tissues (Figs. 3B and 6A), supporting the notion that rBCG-*Ag85A* promotes coordinated activation of immune and metabolic pathways.

Given that *mTOR* has emerged as a central integrator of these signals, increased expression of this gene may potentially reflect mechanisms involved in T-cell differentiation, activation, and effector function (Waickman and Powell 2012). These findings are consistent with previous studies demonstrating that intratumoral BCG immunotherapy induces an efficient antitumor response in murine melanoma in a Myeloid differentiation primary response 88 (*MyD8*8) dependent manner, promoting intense recruitment of inflammatory cells and polarization of the tumor microenvironment toward a “hot” antitumoral profile (Borges et al. 2024). Similarly, Qin et al. (2024) reported that intratumoral administration of high-affinity Ag85A peptides derived from BCG enhanced antitumor efficacy in PPD-positive melanoma. Corroborating these findings, Tarrant et al. (2004) showed that B16-F10 tumors expressing *Ag85A* exhibited inflammatory leukocyte infiltrates detected by hematoxylin and eosin staining, whereas this infiltrate was absent in Hsp65-transfected tumors. Additionally, Wang et al. ( 2023b) demonstrated that Ag85A/B DNA vaccination, administered either intramuscularly or by electroporation, significantly upregulated immune-related pathways and restored metabolic alterations and tissue damage caused by *Mycobacterium tuberculosis* on the mouse. Collectively, these data suggest that transient *Ag85A* expression mediated by rBCG enhances local immune activation and promotes antitumoral effector mechanisms, reinforcing the potential of this prophylactic immunization strategy against cutaneous malignant melanoma.

Overall, these results demonstrate that rBCG exerts a multifaceted antitumor effect, integrating systemic immune activation, modulation of tumor apoptosis, oxidative stress, and pro-inflammatory signaling, which together contribute to exhibit superior efficacy compared to parental BCG in controlling tumor growth. As an initial exploratory study, this work prioritized a comprehensive molecular screening via RT-qPCR to identify the transcriptional pathways modulated by rBCG-*Ag85A* within the tumor microenvironment. This enhanced therapeutic potential was closely associated with a robust activation that orchestrated a potent antitumor immune response. Future studies will expand upon these preliminary molecular findings by utilizing flow cytometry to characterize the specific immune cell populations (such as CD4⁺, CD8⁺ T cells, macrophages, and NK cells) recruited and activated at the tumor site, as well as exploring combination strategies to further enhance antitumor efficacy, with the aim of improving preventive vaccine strategies against melanoma.

## Conclusion

In summary, our study demonstrates that rBCG exhibits superior antitumor efficacy compared to parental BCG, effectively controlling tumor growth and improving survival in B16F10 melanoma models. This enhanced effect is mediated by robust activation of the immune system, including upregulation of apoptosis, inflammatory, and oxidative stress pathways. These findings highlight the potential of rBCG, particularly rBCG-*Ag85A*, as a promising prophylactic vaccine candidate against melanoma. Future research should aim to identify the specific immune cell populations driving these effects and explore combination therapies to further enhance antitumor immunity, ultimately improving preventive immunization approaches for melanoma protection.

## Data Availability

No datasets were generated or analysed during the current study.
